# Telomerase and Telomeres Biology in Thyroid Cancer

**DOI:** 10.3390/ijms20122887

**Published:** 2019-06-13

**Authors:** Benedetta Donati, Alessia Ciarrocchi

**Affiliations:** Laboratory of Translational Research, Azienda Unità Sanitaria Locale-IRCCS di Reggio Emilia, 42123 Reggio Emilia, Italy; alessia.ciarrocchi@ausl.re.it

**Keywords:** telomeres, telomerase, thyroid cancer, *TERT* promoter, EMT, BRD4, epigenetic drugs, combined therapies

## Abstract

Telomere and telomerase regulation contributes to the onset and evolution of several tumors, including highly aggressive thyroid cancers (TCs). TCs are the most common endocrine malignancies and are generally characterized by a high rate of curability. However, a small but significant percentage develops distant metastasis or progresses into undifferentiated forms associated with bad prognosis and for which poor therapeutic options are available. Mutations in *telomerase reverse transcriptase* (*TERT*) promoter are among the most credited prognostic marker of aggressiveness in TCs. Indeed, their frequency progressively increases passing from indolent lesions to aggressive and anaplastic forms. *TERT* promoter mutations create binding sites for transcription factors, increasing *TERT* expression and telomerase activity. Furthermore, aggressiveness of TCs is associated with *TERT* locus amplification. These data encourage investigating telomerase regulating pathways as relevant drivers of TC development and progression to foster the identification of new therapeutics targets. Here, we summarize the current knowledge about telomere regulation and TCs, exploring both canonical and less conventional pathways. We discuss the possible role of telomere homeostasis in mediating response to cancer therapies and the possibility of using epigenetic drugs to re-evaluate the use of telomerase inhibitors. Combined treatments could be of support to currently used therapies still presenting weaknesses.

## 1. Introduction

Telomere protection and/or elongation are considered among the primary mechanisms of cancer survival and aggressiveness. Telomeres, shortening at each mitosis, sense somatic cells aging and induce cell death when they become critically short. Thus, progressive reduction of telomere length is a rate-limiting step for uncontrolled and unlimited cell proliferation capacity. Telomerase is the enzyme responsible for telomere maintenance. It is a ribonucleic complex that includes the telomerase reverse transcriptase (TERT) and its RNA template, the Telomerase RNA component (TERC) [1]. Telomerase is selectively expressed in cells that undergo indefinite proliferation, such as staminal, embryonic and cancer cells. Conversely, cell commitment and differentiation requires telomerase inactivation. Still, mechanisms of telomere maintenance are required in differentiated healthy cells to avoid chromosomes crisis preventing the activation of DNA damage response (DDR) and preserving genomic stability. Thus, telomere homeostasis and telomerase access to telomeres are controlled by an intricate system of accessory protein complexes. Among these, the Shelterin complex is a group of six-proteins, including Telomere repeat factor-1 (TRF1) and 2 (TRF2), protection of telomeres-1 (POT1), TRF1 interacting protein-2 (TIN2), repressor/activator protein 1(RAP1) and the POT1-TIN2 organizing protein (TPP1), highly conserved during evolution. These proteins, positioned on the telomeric loop (t-loop), play a fundamental role in the homeostasis and stabilization of telomeres ends independently from the mechanisms involved in telomere elongation [2]. TIN2 is a bridging molecule that facilitates the assembly of the entire complex. By binding both to double (TRF1, TRF2) and single (POT1) strand DNA, Shelterins shield telomeres from inappropriate DNA repair avoiding the activation of DDR pathways and non-homologous end joining (NHEJ). In particular, RAP1 recruited by TRF2 inhibits homologous recombination. Furthermore, Shelterins protect telomeres from end-to-end fusion events and degradation [3]. Finally, TPP1 functions by regulating telomerase access to telomere, and the entire complex regulates nucleosomes distribution and contributes to the establishment of an epigenetic environment prone to telomere maintenance [4,5].

Being the sensor of cell proliferative capacity, aberrant telomere elongation occurs in the majority of cancers due to telomerase reactivation (in 90% of malignancies) and/or alternative lengthening of telomeres (ALT) [6]. Alteration in *TERT* expression and/or activity has been reported in many cancers and associated with increased aggressiveness. Among the mechanisms leading to aberrant telomerase activation, *TERT* promoter mutations C228T and C250T (−124 bp and −146 bp upstream of *TERT*’s transcription starting site, respectively) are frequent in cancer and associated with increased aggressiveness and metastatic potential in several tumor settings. These alterations cause the formation of ectopic binding sites for specific transcription factors (TFs) within the *TERT* promoter inducing its overexpression [7]. Similarly, aberrant expression and/or mutations of Shelterin proteins can favor tumorigenesis either by induction of chromosomal rearrangements and genome instability and by contributing to alteration of telomere length [8]. ALT occurs often in cancer that do not express telomerase, as the consequence of recombination-dependent replication pathways of telomere extension. ALT activation mainly correlates with the presence of mutations in *ATRX chromatin remodeler* (*ATRX*) and *death domain associated protein* (*DAXX*) genes in both tumors and cell lines [9,10,11,12]. In several tumors, mutations in the *TERT* promoter and *ATRX* have been reported to be mutually exclusive. However, melanomas with loss-of-function mutations in *ATRX* have also been found to have *TERT* promoter mutations [11,13].

Thyroid cancers (TCs) are the most common endocrine malignancy. Metastasis and aggressive clinical behavior in these tumors are generally limited to a small but significant group of high-grade lesions (about 10% of all diagnosed thyroid tumors) with poor biological characterization and very limited therapeutic options [14]. These tumors, associated with poor prognosis and low rate of survival, comprise a range of highly heterogeneous diseases that includes well differentiated TCs that developed Distant Metastases (DMs), Poorly Differentiated Thyroid Cancers (PDTCs) and Anaplastic Thyroid Cancers (ATCs). Clinical management of TC patients encompasses a spectrum of options, ranging from sonographic surveillance to total thyroidectomy plus lymph node neck dissection and/or radioiodine ablation (RAI). The lack of reliable markers for predicting the aggressive behavior of these tumors is a severe limitation to a precise risk-based stratification of this disease, thus contributing to the issue of patients’ overtreatment [15]. Furthermore, over 75% of high-grade TCs are radioiodine refractory and no effective therapies are available. These patients represent a relevant clinical challenge [16]. As for other tumors, TCs heavily rely on aberrant telomere regulation. In this review, we aim to summarize the current knowledge about telomere regulation in TC, with a particular focus on highly aggressive lesions and with the intent to discuss the potential application of these observations for defining both new prognostic tools and new potential targets to improve patient management. 

## 2. *TERT* Promoter Mutations Are a Hallmark of TC Aggressiveness

Mutations in the *TERT* promoter are strongly over-represented in highly aggressive TCs, with the C228T usually being more frequent than C250T [17,18,19,20]. Their presence strongly correlates with disease stage III/IV, metastatic behavior, cancer recurrence and reduced survival probability [15,17,21,22,23]. A recently published study, conducted on data from The Cancer Genome Atlas (TCGA) consortium, revealed that TCs harboring *TERT* promoter mutations consistently present distinct transcriptomic profiles associated with enhanced cell cycle progression and metabolic activities as compared to wild-type patients [24]. As in other settings, also in thyroid, *TERT* promoter mutations are markers of malignant transformation, since no evidence of their occurrence in benign thyroid lesions has been reported so far. Furthermore, *TERT* promoter mutation frequency increases dramatically from low-grade microcarcinomas (where they were reported in fewer than 5% of lesions [25]) to highly aggressive poorly differentiated and undifferentiated TC (48.8% and 41.8%, respectively) independently of the ethnic and geographical backgrounds of the studies. In well-differentiated papillary thyroid cancers (PTCs), *TERT* promoter mutations are markers of metastatic behavior. Indeed, while in non-metastatic lesions *TERT* mutations are detected in about 10% of cases, 33% of PTCs that develop distant metastasis harbor mutations in the promoter of *TERT* gene [23], highlighting a role of these alterations in conferring aggressiveness to PTCs. Furthermore, De Biase et al., with the intent of exploring potential commonalities, compared the genetic background of 29 DMs and 18 ATCs. They showed that these highly aggressive thyroid lesions are characterized by different genetic backgrounds and likely evolve through different molecular mechanisms with the sole exception of *TERT* promoter mutations, which were the only high-frequency genetic events shared between these two separate groups of TCs. Together, these observations define the presence of *TERT* promoter mutations as a unique and reliable feature of aggressive TCs and suggest these genetic alterations as candidates for being a potential prognostic tool for identifying aggressive forms of TC at diagnosis [26]. In line with this, *TERT* expression and telomerase activity are detected in thyroid carcinoma, but not in normal thyrocytes or in benign thyroid tumors [17]. Interestingly, in the vast majority of published studies, *TERT* promoter mutations correlate with *B-Raf proto-oncogene, serine/threonine kinase* (*BRAF*) V600E mutation, considered a major oncogenic determinant of TC. Patients harboring both *BRAF* V600E and *TERT* promoter mutations display more severe clinical factors, suggesting that these mutations contribute to different molecular mechanisms resulting in synergic effects [17,18,27]. Additionally, a significant association was also observed between *TERT* promoter mutations and *RAS* mutations, which are frequent in PDTCs and ATCs [17,19,21]. To further strengthen the role of TERT in high-grade TC, our group identified the first genetic signature distinctive of DMs and significantly associated with increased mortality. This signature, called THYT1, includes, in addition to Ch1q duplication, not only *TERT* promoter mutations, but also duplication of *TERT* locus on chromosome 5 [15]. These data highlight the importance of *TERT* locus in the progression of TC. Furthermore, THYT1 may represent a prognostic marker to improve risk-based stratification and management of pre-operative PTCs.

Differently from the vast majority of cancer driver mutations that fall within the coding region of oncogenes and/or tumor suppressors altering the resulting proteins, *TERT* promoter mutations occur within non-coding elements, and their mechanism of action has only partially been elucidated. Both of the most frequent mutations (C228T and C250T) introduce within the *TERT* promoter new binding sites for members of the E-Twenty-Six (ETS) family of transcription factors. The increased activity of these TFs enhances *TERT* expression, leading to an augmented telomerase activity [28]. Experiments of site-direct mutagenesis recently conducted in glioblastoma cell lines confirmed the high specificity of the ETS consensus sequence necessary for *TERT* transcription activation. Different substitutions in the same position like G228C, G250C or G250T showed no effect on *TERT* transcription. In contrast, the G228T mutation partially induced promoter activity, being collocated in a position of ETS motif frequently degenerated for A/T. To identify which among the ETS transcription factors was responsible for *TERT* activation, a small interfering RNA screening of 13 molecules was conducted. The GA-binding protein alpha subunit (GABPA) KD resulted in a substantial decrease in *TERT* expression. Consistently, only GABPA KD impaired mutant promoter activity, despite having no effect on the wild type sequence in Luciferase assay indicating that GABPA is the only ETS factor able to affect *TERT* expression in presence of cancer-associated mutations [28]. The selective binding of GABPA to mutant promoter was confirmed in vivo and extended to different tumor settings by the analysis of ChIP-seq data from ENCODE project [29]. In contrast, Yuan et al. observed an inverse correlation between GABPA and TERT expression in primary TCs, where lower GABPA expression was associated with aggressive disease and reduced overall survival. These observations propose a potential tumor suppressor activity of GABPA. According to the model suggested by these authors, GABPA exerts a potent anti-metastatic function by directly stimulating the expression of Dicer1, a master suppressor of cancer initiation and progression. This discrepancy with regard to the role of GABPA as both promoter and suppressor of cancer aggressiveness warrants further investigation [30].

The methylation status of the chromatin surrounding *TERT* promoter is also affected by the presence of cancer-associated mutations reflecting the transcriptional activity of this region. The mutant *TERT* promoter exhibits high levels of H3K4me2/3, markers of opened chromatin and low CpG methylation upstream of the transcription starting site. On the contrary, high levels of H3K27me3 and CpG methylation outline wild-type *TERT* promoter, indicating a more silenced transcriptional state [31]. Recently, Liu R et al. proposed a model to explain the synergistic effects of *TERT* promoter mutations and *BRAF* V600E that perfectly fits in this context. It is known that binding of GABP to mutant *TERT* promoter, necessary for *TERT* activation, is influenced by the presence of native ETS motif spanning the two mutations and allowing the binding of tetramers of GABPA and GABPB. These authors demonstrated that *BRAF* V600E enhances the activation of Mitogen-activated protein kinase (MAPK) pathway, leading to the FOS-mediated expression of GABPB, which, in turn, binding to *TERT* mutated promoters in tetrameric form, induces *TERT* overexpression [32]. In agreement with this, a recent report showed that *TERT* expression in TC patients is positively correlated with both *TERT* promoter and *BRAF* V600E mutations. Mechanistically, this was due to the BRAF-induced upregulation of the PEA3 subfamily of ETS transcription factors (ETV1, ETV4 and ETV5), which in turn bind and selectively activate the mutant promoter driving *TERT* expression [33].

Ankicilar et al. investigated how *TERT* promoter mutations drive *TERT* activation and, by circular chromosome conformation capture (4C) assay, they demonstrated that GABP mediates a long-range chromatin interaction that along with an enrichment of active histone marks consequently drives *TERT* transcription. Indeed CRISPR/CAS9-mediated reversal of *TERT* promoter mutations to the wild type residue abrogates this long-range chromatin interaction and RNA Polymerase II recruitment, and reduces *TERT* transcription and telomerase activity. Furthermore, they found that a major player of this complex assembly is Bromodomain-containing protein 4 (BRD4), which is able to interact with the long-range region 300kb upstream of mutant *TERT* promoter enhancing *TERT* expression. Accordingly, BRD4 KD led to significant loss of active histone marks and impaired long-range chromatin interaction to an extent similar to GABPA KD. Furthermore, BRD4 regulates *GABPA* transcription by directly binding its promoter [34]. BRD4 belongs to the BET family of proteins, it is able to bind acetylated lysines, and it has both acetyl-transferase and kinase activities. It promotes cell cycle progression and cell growth in cancer. In particular, a recent study demonstrated a direct role of BRD4 in ATCs, where its silencing significantly inhibited tumor growth both in vitro and in vivo [35]. Furthermore, it has been already demonstrated in mouse models that the employment of BET inhibitors (BETi), a new class of anticancer drugs design to block the activity of BRD4 and the other BET proteins, is able to suppress ATC growth and to improve survival [36]. In line with this, Wang et al. showed that treatment with different kinds of BETi blocks telomere elongation and Telomerase complex activity [37]. Thus, this evidence suggests that telomere alterations in aggressive TCs may rely on BRD4 activity, prompting the use of BETi already employed in clinical trials for several tumor settings. Thus, the use of BETi may represent an alternative way to counteract telomeres deregulation avoiding side effects of direct telomerase inhibitors. Furthermore, the employment of BETi may present a selective strategy for targeting cancer cells presenting *TERT* promoter mutations sparing the ones that are not affected by these mutations, including normal cells. 

## 3. Additional Mechanisms of Aberrant *TERT* Expression Regulation in Cancer

*TERT* promoter contains binding sites for several known regulatory factors, which include GC-motif and E-boxes. It has been widely demonstrated that MYC proto-oncogene, bHLH transcription factor (c-Myc) directly regulates *TERT* expression by binding to E-boxes within the *TERT* promoter [38]. Several studies have described c-Myc activation in TCs and its association with features of aggressiveness [39,40]. Still, whether and to which extent telomere deregulation relies on c-Myc activity is not well defined. Interestingly, the *BRAF* V600E - MAPK pathway axis may activate *TERT* expression through c-Myc in a *TERT* promoter mutation-independent manner, even if this mechanism seems to be less robust than that previous cited in *TERT* promoter mutated lesions [32]. c-Myc alone is likely not sufficient to drive *TERT* expression and the cooperation with Specificity Protein 1 (Sp1), a transcription factor that binds GC-motifs next to E-boxes, seems to be required for an efficient *TERT* promoter activation. Intriguingly, BETi, which we previously described as a potential therapeutic options for aggressive TCs harboring *TERT* promoter mutations, have been shown to drastically suppress *c-Myc* expression, and this effect has been widely documented in human patients in several clinical studies for solid and hematological tumors [41,42]. Thus, the employment of these drugs in aggressive TCs could exert a double activity, directly blocking the effect of BRD4 on *TERT* promoters and indirectly by restraining the expression and transcriptional activity of c-Myc on *TERT* regulation. 

It has been reported that the nuclear factor kappa B (Nf-KB) pathway is able to enhance *c-Myc* and *Sp-1* gene expression driving *TERT* activation in several hematological cell lines [43,44]. Furthermore, an NF-κB-responsive element is present in the *TERT* promoter 600 bp upstream of the transcription starting site [45]. However, in TCs, NF-κB signaling may have marginal effect on aggressiveness [46]. Indeed, a recent study used different NF-κB targeting strategies (genetics and pharmacologic) in different TC cell lines to demonstrate that NF-κB inhibitors are unlikely to be beneficial, even in combination with chemotherapy or radioiodine therapy [47]. 

*TERT* promoter also presents binding sites for signal transducer and activator of transcription 3 (STAT3), located about 2000 bp upstream of the transcription starting site. Published studies have suggested that STAT3, a TF activated in response to various cytokines and growth factors, in TC may function as onco-suppressor rather than as cancer promoting factor. Indeed, although *STAT3* expression is detected in PTCs [48], the majority of the studies reported an increased activity in normal tissues compared to cancer lesions and an inverse correlation between *STAT3* expression and tumor size [49,50,51]. 

Paired box 8 (PAX8) is an important TF in thyroid tumorigenesis. Additionally, it is known to be a marker of thyroid cell differentiation; PAX8 is among the more reliable markers of thyroid specification in advanced TCs, and indeed its expression has been detected in biopsies of ATC patients [52]. PAX8 responsive elements are found in *TERT* promoters, and it has been demonstrated that this TF positively regulates the expression of *TERC* raising the hypothesis that it plays a role in telomere regulation [53]. 

Recently, we described a relevant role for activator protein-1 (AP1) in driving thyroid tumorigenesis by controlling the expression of the pro-oncogenic factor *Runt-related transcription factor 2* (*RUNX2*). AP-1 is a leucine zipper heterodimeric complex and the Jun-Fos dimer is the most common form of AP-1 protein in human cells. We reported the collaboration of c-JUN and BRD4 in the regulation of *RUNX2* where they orchestrate the cooperation of three distal active enhancers (ENHs) with the *RUNX2* promoter. Two hypotheses for their interplay have been formulated: either BRD4 binding to acetylated regions may function as a docking site for the recruitment of c-JUN on *RUNX2* regulatory regions or c-JUN binding to ENHs favors BRD4 recruitment [54]. A similar mechanism of cooperation between these two factors can occur also on *TERT* promoter. Indeed, AP-1 can directly bind to the *TERT* promoter through binding sites at positions −1655 and −718 [55]. Additionally, BRD4 has been reported to interact with the mutated form of the *TERT* promoter by long-range chromatin interaction, the same ability BRD4 has to modify chromatin structure observed in the *RUNX2* locus [34]. However, AP-1 recruitment at *TERT* promoter seems to have an inhibitory effect on *TERT* expression. Consistently, the mutations at both AP-1 binding sites eliminated about 70% of the suppressive effect caused by AP-1 [55]. Contrasting evidence links *AP-1* expression to TC features in vivo. Some reports indicate that the expression of *AP-1* was negative correlated with tumor size in PTCs [56]. Conversely, in a very recently published work, the level of AP-1 protein was found to be significantly up-regulated in PTCs compared with surrounding normal thyroid tissue by immunohistochemistry and was positively correlated with tumor size [57]. Thus, it remains to be established whether the activity of AP-1 in favoring TC progression partially relies on impairing telomere homeostasis and if yes with which mechanisms. 

A comprehensive description of *TERT* promoter consensus sequences has been reviewed by Ramlee and colleagues, and may be used as a further hint to study possible players in telomerase regulation in aggressive TCs [45].

## 4. Telomere Length Involvement in Thyroid Cancer Development

In the spectrum of thyroid tumors, familial forms of non-medullary TCs (NMTCs) have also been reported. Familial NMTC patients show progressively increased disease aggressiveness in subsequent generations. This phenomenon is called “genetic anticipation”, and it was defined and associated with several inherited benign and malignant disorders. In the case of NMTCs, the later generations when compared to the first one showed an earlier age of insurgence, higher rate of lymph node metastases at surgery, and a worse outcome [58,59]. Familial NMTCs are generally more aggressive than the sporadic forms [60] and are associated with a 5–10-fold higher risk of developing the tumor for first-degree relatives compared to the general population. In recent years, several groups have collected and investigated pedigrees of familial NMTC patients in order to find the predisposing genetic loci, but this is probably a complex disease to the onset of which several genetic and environmental factors contribute [59]. Telomere length is a strong hereditable tract of these patients, and progressive telomere shortening through generations may represent the basis of anticipation. In line with this, anticipation is a typical condition of familial telomere diseases, a spectrum of genetic degenerative and age-dependent disease caused by premature senescence of stem cell compartments and determining increased rates of organ failure and cancer [61]. Indeed, short telomeres induce genomic instability and activate pathways of DNA damage response, whose functionality is crucial for cancer evolution [62]. The presence of short telomeres is reported in the blood of patients affected by different types of cancers, such as head and neck, bladder, lung, renal and breast cancer [63,64], enforcing the hypothesis that telomere dysfunction may represent a risk factor for cancer development. Confirming these data, familial PTCs also display short telomeres in peripheral leukocytes [63,65]. Interestingly, unaffected familial members present telomere lengths similar to those of relatives affected by PTC, suggesting that they likely have a genetic background prone to cancer development and probably need a lower number of additional factors to achieve tumor onset compared to sporadic cases [66]. Cantara and colleagues reported similar evidence in phytohemagglutinin-stimulated T-lymphocytes of both sporadic and familial PTC patients in which shorter telomeres with respect to healthy subjects were observed. Despite short telomeres, high telomerase activity and *TERT* expression were observed in PTCs as in other human cancer tissues [67,68]. Thus, telomerase activation is probably necessary as a telomere maintenance mechanism, likely not for inducing extensive telomere lengthening, but in order to conserve the minimal telomere length indispensable to save DNA-damaged cells from apoptosis, therefore contributing to their genomic instability and immortalization. Consistently, study on familial PTCs has demonstrated that these patients are characterized by abundant acentric fragments presenting telomeric sequences derived from subtelomeric chromosomal breaking, and thus, they show a frequency of chromosome fragility higher than healthy subjects and sporadic PTCs [66]. Overall, we can speculate that short telomeres are predisposed to genomic instability, and hence the occurrence of cancer-driving mutations, which include *TERT* promoter mutations [69,70]; subsequently, telomerase activation maintains telomere length and sustains cancer evolution and progression.

## 5. Telomere-Independent Cancer Supportive Mechanisms of TERT

Telomere-independent activities of TERT have been reported, and several studies have shown that this protein is engaged in processes such as regulation of DNA damage response, repression of apoptosis, regulation of chromatin state, and enhanced cell proliferation [71,72,73]. Furthermore, mounting evidence indicates a role of TERT in supporting metastatic spreading of cancer by the activation of the epithelial to mesenchymal transition (EMT) [74,75]. EMT is a process which allows epithelial cells to transiently transdifferentiate and acquire a mesenchymal-like phenotype. During this transition, epithelial cells lose their differentiated characteristics, including cell–cell adhesion, polarity, and lack of motility, and acquire mesenchymal features, including motility, invasiveness, and resistance to apoptosis [76,77]. Activation of the EMT program is triggered by many signaling factors and extracellular stimuli among which transforming growth factor beta (TGF-β) is considered a master player. Once engaged on its target receptors, TGF-β signals act within the cells through three major pathways: (1) inducing nuclear translocation of the SMAD family member 2/3 (SMAD2/3) proteins, (2) activating the MAPK, and (3) Phosphatidylinositol-4,5-Bisphosphate 3-Kinase—AKT serine/threonine kinase 1 (PI3K-AKT), signaling cascades. These pathways converge on the regulation of several TFs that together orchestrate gene expression silencing of epithelial-associated genes (like epithelial cadherin and cell–cell adhesion molecules) while promoting the expression of mesenchymal genes (including mesenchymal-cadherins, cell-matrix interacting molecules and cytoskeleton filaments) [78]. In addition to inducing migratory and invasive capacities, EMT also allows cancer cells to acquire higher resistance to apoptosis and immunosuppression. EMT is also crucial in TC, where it has been suggested to play a function both in the transient transdifferentiation of well-differentiated lesions during the metastatic spreading, as well as in the constitutive de-differentiation of highly aggressive ATCs. We recently reported that in PTCs, Cadherin 6 (CDH6), a class II cadherin, is induced by TGF-β and promotes the activation of EMT by restraining autophagy [79,80,81]. Strikingly, we also reported that *CDH6* expression in human PTCs is restricted to highly metastatic lesions, in which we also found a significantly higher incidence of *TERT* promoter mutations and *TERT* locus amplification, raising the hypothesis of a functional interconnection between these molecular events [15,23]. Indeed, evidence exists that TERT takes part in the activation of EMT and this activity is independent by its function in maintaining telomere length [75]. 

Liu et al. reported that overexpression of *TERT* promotes the acquisition of mesenchymal features in gastric cancer. Conversely, its knockdown causes loss of mesenchymal markers and reduced invasiveness. In this model, TERT mediates the TGF-β signaling and in TERT KD cells TGF-β induction of target genes expression is impaired. Indeed, TERT interacts with beta-catenin (one of the EMT associated TFs and mediator of the Wnt-signaling) and together associate with mesenchymal marker promoters to drive their expression. This effect was largely independent of the telomere-lengthening activity of TERT, since overexpression of *TERT* mutants lacking these properties are still able to induce expression of EMT associated genes [75]. The transcriptional activity of TERT and its role in EMT are consistent in other models. Qin and colleagues reported that TERT promotes EMT in colon cancer through the Zinc finger E-box binding homeobox 1 (ZEB1) pathway. ZEB1 is a TGF-β target and a master regulator of EMT. The authors showed that TERT forms a complex with ZEB1 which binds to *E-Cadherin* promoter and represses its transcription leading to EMT [82]. The activity of TERT in this context is ZEB1 dependent, since overexpression of TERT fails to repress E-Cadherin in cells where ZEB1 expression is compromised. Intriguingly, EMT-related TFs seem to feedback on telomere homeostasis in a positive feedback loop. Snail Family Transcriptional Repressor 1 (Snail1) is an EMT TFs that belongs to the zinc finger TFs family. The relevance of Snail1 in supporting EMT is highlighted by a large amount of evidence. Recently, a role of Snail1 in controlling telomere integrity has been proposed. Mazzolini et al. reported that Snail1-depleted mesenchymal stem cells show a dramatic increase in telomere alterations and a significant reduction of telomere length. However, activity of telomerase is higher in Snail1-depleted cells, as is the expression of TERRA, a telomere-associated long non-coding RNA [74]. TERRA is a highly heterogeneous family of transcripts transcribed from subtelomeric regions. Loss of function as well as de-localization of TERRA leads to severe telomere dysfunction indicating that both expression and localization of this lncRNA are strictly necessary for telomere maintenance [83]. In the context of mesenchymal stem cells, *Snail1* overexpression leads to a strong inhibition of TElomeric Repeat-containing RNA (TERRA) and *TERT* expression, coherently with the increased telomerase activity observed in *Snail1*-depleted cells [74]. In particular, the observation that Snail1 directly controls TERRA identified this TF as one of the factors controlling transcription at telomeric regions. TERRA expression is repressed by TGF-β concomitantly with *Snail1* upregulation. Furthermore, RNA-sequencing profiling in a TERRA overexpressing model of mammary epithelial cells, showed that TERRA impaired TGF-β signaling by altering the response of a large number of EMT-related genes to the TGF-β stimulation. Together, these data indicate that expression of TERRA prevent activation of EMT and that the Snail1-mediated inhibition of this lncRNA is required to drive EMT [74].

## 6. Are Telomeres Possible Targets for TC Therapy?

For many years, therapeutic options for TC patients were limited to surgery and radioiodine ablation (RAI). However, 15–20% of well-differentiated cases that generate distant metastasis, as well as most of ATCs, remain resistant to standard treatments or present contraindication for surgery [84,85]. 

However, in recent years, several new drugs have been approved for the use in TCs. In this section, we intend to discuss some of these new options and their possible interplay with telomere biology. Mutations in *BRAF* and other members of the MAPK pathway are frequently mutated in TCs. *BRAF* V600E specific (Vemurafenib) and BRAF-MEK dual inhibitors (Dabrafenib) were recently proposed as potential therapies for advanced forms of TC, including ATCs [86,87]. Furthermore, multiple kinase inhibitors (MKIs), like Sorafenib and Lenvatinib, were FDA approved for the treatment of RAI-refractory and/or recurrent TCs. Both of these drugs improved overall survival compared to placebo in phase III randomized clinical trials; however, their clinical benefits were lessened by induced systemic toxicity [88,89,90,91,92]. As previously discussed, MAPK signaling converges on the activation of many cancer-related TFs, including AP-1 and c-Myc [93,94]. Both these TFs were proposed as potential regulators of *TERT* promoter, and therefore the use of these drugs may impact *TERT* expression and consequently the biology of telomeres [38,55].

Immunotherapy, aiming to exploit immune response and restore its ability to eliminate tumor cells, represents one of the more recent frontiers for tumor treatment. Among others, Pembrolizumab, targeting PD-1 by avoiding the interaction with its ligand, was tested in a clinical trial involving different solid malignancies including thyroid tumors [95,96]. This approach seems promising since recently published data showed that the 70–90% of highly aggressive TCs are positive for PD-L1 expression with respect to normal thyroid and differentiated tumors resulting negative [97]. However, the clinical benefits of these drugs need to be maximized, and therefore, several clinical trials are testing the effects of combined therapies for advanced tumors. Among others, some trials are currently testing the interaction between immunotherapy and epigenetic drugs, including one investigating the relationship between BETi and PD1/PD-L1 signaling blockade [98]. In line with this, a recent study conducted in a mouse model of c-Myc driven lymphoma showed that simultaneous inhibition of the PD-1/PD-L1 axis and treatment with the BETi JQ1 caused synergistic responses, demonstrating an important functional interaction between BETi and the host immune system [99]. For a more complete overview of the studies conducted up to date, Naoum and colleagues provided a detailed list of all clinical trials currently being developed for the treatment of highly aggressive TCs [85]. The observations collected in this review pave the way for new interesting speculations for the employment of alternative combined treatments. Solid evidence indicates that cancers with a high degree of genomic damage e/o mutational load are more sensitive to immunotherapy [100,101,102]. The reasons for this phenomenon are still to be determined, but the implications of these observations are already under investigation in clinics. Noticeably, aberrant telomere regulation may affect genomic stability by increasing DNA damage and consequently may condition immunotherapy response in cancer. Furthermore, Telomerase-based immunotherapy has also been developed and relies on the possibility of using TERT-derived peptides processed and presented on the surface of cancer cells as cancer neo-antigens, stimulating immune-mediated cell-kill. Two approaches have been proposed: one based on immune activation in vivo through the direct injection of optimized TERT derived peptides, and the other ex vivo based on the activation and expansion of patient-derived dendritic cells and then transfected with peptide and re-administered to patients through intradermal injection. For both of these strategies, clinical trials are ongoing in different tumor settings, and preliminary results have shown good tolerability and encouraging responses [103,104,105,106,107,108]. Recent evidence demonstrates that combination of checkpoint inhibitors with traditional cancer vaccines enhances anti-tumor activity in some studies and improves response to immunotherapy, laying the basis for a promising use of Telomerase vaccines in combination with immunotherapy [109]. Further studies are needed to strengthen the link between *TERT* regulation and immunotherapy response. However, in this context, telomere biology should be exploited both as potential biomarker for response as well as potential co-target to improve immunotherapy response in combination therapy schedules. 

Targeting telomerase and mechanisms regulating telomere homeostasis may be considered a promising anti-cancer strategy. However, drugs directly targeting the telomerase complex have shown effectiveness in preclinical studies, but in clinical trials show lower performance and high side effects of toxicity [110,111,112,113]. In this context, the possibility of counteracting telomere impairment with alternative strategies appears to be of great interest. Here, we propose the employment of BETi drugs to indirectly target telomerase [114]. Inhibiting BRD4 transcriptional activity through the use of BETi may directly reduce *TERT* expression, but may also indirectly condition its transcription by repressing other BRD4-dependent TFs like *c-Myc*. Furthermore, the involvement of BRD4, as well as HDAC (which also controls histone acetylation at the telomere level), in telomere regulation suggests the use of epigenetic drugs (such as BETi and HDAC inhibitors) to improve the effectiveness of telomerase-inhibiting compounds. Both of these drugs, when employed alone, failed to show substantial effects in clinical trials [115,116]. However, their use in combination with other compounds showed promising results [117]. We may intriguingly speculate as to the use of BETi and HDACi in combination with telomerase inhibitors. Their effects on *TERT* expression and telomeres regulation may synergize with the one of telomerase inhibitors and improving their effectiveness. This could potentially allow the use of telomerase inhibitors at lower doses, thus limiting the toxicity of these drugs.

## 7. Conclusions

**Figure 1:**
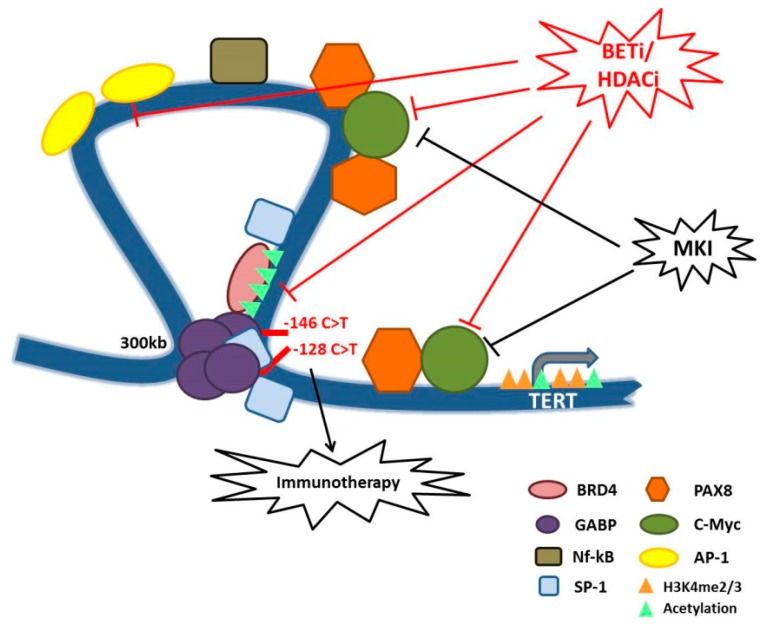
Schematic model representing *TERT* promoter regulation in TCs and some hypotheses of therapeutic interventions. The figure shows the long-range chromatin interaction mediated by GABP factors and BRD4 on the mutated *TERT* promoter, and all the transcription factors positively influencing *TERT* expression and for whom we described a possible connection with *TERT* promoter in TCs. We also summarized the new proposed therapies for aggressive TCs, that may also rely on telomerase inhibition. In particular, MKI that acts on MAPK signaling and epigenetic drugs such as BETi and HDACi that may counteract *TERT* expression by impairing the function of TFs such as c-Myc and BRD4. Moreover, telomeres deregulation and the subsequent genomic instability, here represented by *TERT* promoter mutations, may influence the response to immunotherapeutic treatments. Overall, this model shows that there are many possible therapeutic combinations to counteract the aberrant activity of telomerase in TCs and consequently to inhibit one of the major trigger of TC aggressiveness.

TC represents a paradigmatic example of the importance of telomere regulation in malignancies. Currently, mutations in *TERT* promoter are among the more common genetic abnormalities observed in cancers and the most frequent alterations observed in aggressive thyroid tumors. Mutations in *TERT* promoter are generally associated with increased *TERT* expression and Telomerase activity and although they are not always connected to increased telomere length [118], they are close related to bad prognosis, metastatic spreading and low patients overall survival [15,17,21,22,23,43,44,59,76,106]. We also explored the wide spectrum of signaling pathways converging on *TERT* promoter and the possible still poorly understood connections with the biological mechanisms already described in thyroid cancer biological settings. Overall, this evidence highlights the feasible employment of BETi for the treatment of thyroid tumors. Indeed, these drugs seem to hamper tumor spreading by different sides and having beneficial effects both in tumor harboring *TERT* promoter mutations and in the wild type ones. Here, we also discussed the available evidence showing the ability of BETi to disrupt long-range chromatin interaction on mutated *TERT* promoter such as to inhibit c-Myc and AP-1 transcriptional activities independently from tumor genetic background (Figure 1) [34,41,42,54].

The role of Shelterin proteins in the regulation of telomeres has not been studied in deep in TC setting. A single paper reported no mutations or polymorphisms in Shelterin complex genes in familial PTCs [119]. However further studies are necessary to define the structural organization of telomere proteins in TC lesions and whether TERT is the major players of telomere regulation or other mechanisms intervene. Conversely, ALT mechanisms as determined by mutations in *ATRX* and *DAXX*, are not reported in non-medullary cells derived thyroid cancers in many different studies [6,11,118], thus an involvement of these mechanisms in telomere deregulation may be likely excluded in this pathogenic environment. 

In conclusion, these data enforce the role of telomerase dependent mechanisms in sustaining TC progression. Thus, telomerase regulation represents a major challenge and an interesting hint for studies investigating the pathogenesis of TC and/or the employment of new combined treatments.
